# Extended spider cognition

**DOI:** 10.1007/s10071-017-1069-7

**Published:** 2017-02-07

**Authors:** Hilton F. Japyassú, Kevin N. Laland

**Affiliations:** 1 Biology Institute, Federal University of Bahia, Rua Barão de Jeremoabo s/n, Campus de Ondina, Salvador, Bahia 40170-115 Brazil; 2 Centre for Biodiversity, School of Biology, University of St Andrews, Harold Mitchell Building, St Andrews, Fife UK KY16 9TH

**Keywords:** Extended cognition, Modular cognition, Web building, Evolvability, Niche construction

## Abstract

There is a tension between the conception of cognition as a central nervous system (CNS) process and a view of cognition as extending towards the body or the contiguous environment. The centralised conception requires large or complex nervous systems to cope with complex environments. Conversely, the extended conception involves the outsourcing of information processing to the body or environment, thus making fewer demands on the processing power of the CNS. The evolution of extended cognition should be particularly favoured among small, generalist predators such as spiders, and here, we review the literature to evaluate the fit of empirical data with these contrasting models of cognition. Spiders do not seem to be cognitively limited, displaying a large diversity of learning processes, from habituation to contextual learning, including a sense of numerosity. To tease apart the central from the extended cognition, we apply the mutual manipulability criterion, testing the existence of reciprocal causal links between the putative elements of the system. We conclude that the web threads and configurations are integral parts of the cognitive systems. The extension of cognition to the web helps to explain some puzzling features of spider behaviour and seems to promote evolvability within the group, enhancing innovation through cognitive connectivity to variable habitat features. Graded changes in relative brain size could also be explained by outsourcing information processing to environmental features. More generally, niche-constructed structures emerge as prime candidates for extending animal cognition, generating the selective pressures that help to shape the evolving cognitive system.

## Boundaries of cognition

The field of animal cognition is subject to several controversies, perhaps the most obvious of which is the tension between a conception of cognition as operating like a general purpose problem-solving device and the view that cognition is more reliant on functionally dedicated, specialised neural modules. The former position remains influential within experimental psychology, while the latter is prevalent among evolutionary biologists, behavioural ecologists and evolutionary psychologists (Laland and Brown 2011; Sanderson 2014), although a range of intermediate positions are tenable. What these stances have in common, however, is the premise that, excluding some aspects of perception, cognition primarily occurs within the boundaries of the central nervous system (CNS). A further controversy, potentially even more fundamental than the aforementioned one, takes issue with this premise by extending the seat of cognition into the external environment, either through an extended (Wilson 2008) or enacted (Thompson 2010) cognition thesis, or through a dynamical systems approach (Shanker and King 2002).

Resolution of these tensions requires accurate accounts of alternative conceptions of cognition and their contrasting predictions, together with clear-cut criteria for deciding between them (Kaplan 2012). It is not in the scope of this paper to discuss a definition of cognition in itself, although the findings we present clearly have implications for such a definition. Instead, we embrace a broad and prevalent definition of cognition as the acquisition, processing, storage and use of information (Shettleworth 2010). In doing so, we focus on contrasting the conceptions of cognition that confine cognition to the CNS (which we refer to as the ‘central cognition’ hypothesis—Fodor 1983; Barkow et al. 1995), and those that extend it to the body or its contiguous environment (the ‘extended cognition’ hypothesis), using the cognition of spiders as a model system.

The central cognition hypothesis is being challenged from various perspectives (Noë 2004; Churchland 2007). It is clear, for example, that humans extend their information processing power through computers and a variety of so-called intelligent gadgets, but many other animals use tools, some of which may potentially function as information processing devices (Biro et al. 2013). It remains possible that some animal-constructed devices will also help the constructor to perform cognitively challenging tasks. We make no distinction here between the various ways that animals could extend their cognition out of the brain, nor are we defending one of these particular ways of extending cognition. While we acknowledge that differences exist between the embodied (Varela et al. 1992), the extended (Clark and Chalmers 1998), and the enactive (Thompson 2010) views of cognition, here we concentrate on the general similarity between them: that is, we focus on the general idea that somehow cognition also operates outside the bounds of the brain, and accordingly we refer to all variants of these views as extending cognition.

 The central and extended cognition hypotheses lead to fairly general contrasting predictions. Ashby’s (1960) ‘Law of Requisite Variety’ specifies that, if it is to be stable, the number of states of the control mechanism of a system (e.g., the variant states available to an organism) must be greater than or equal to the number of states in the system being controlled (e.g., the variant environmental states with which the organism must cope). It follows that organisms that experience more complex environments require more complex behaviour. Hence, from a central cognition perspective, individuals inhabiting more challenging environments, for instance with unpredictable fluctuations in resources, should possess larger or more complex brains than those in less complex environments (Shumway 2008). This prediction follows from the assumption that neural complexity or processing power underlies behavioural complexity, and that the requirement to solve challenging environmental problems in order to survive and reproduce selects for a rich neural architecture in the CNS capable of delivering behavioural flexibility. As an illustration of this reasoning, the ‘social brain hypothesis’ states that living in groups is cognitively demanding, because the complexities of social interactions impose a burden on cognitive processing (Dunbar 1998; Dunbar and Shultz 2007), for instance, requiring individuals to remember and contextualise diverse relationships, which is known to be critical for effective social interaction.^1^ Accordingly, living in larger groups is associated with increases in grey matter in mid-superior temporal sulcus and rostral prefrontal cortex in Macaques (Sallet et al. 2011). In general, the social brain hypothesis has found wide acceptance, notwithstanding criticisms concerning, for example, the difficulties it faces in accommodating non-modular aspects of intelligence (Reader et al. 2011) and in explaining graded changes in relative brain size (van Schaik et al. 2012).

Conversely, the extended cognition perspective anticipates a less clear-cut relationship between environmental complexity and brain size. That is because this position does not conceptualise cognition as exclusively grounded in the central nervous system, but instead regards cognition as a more or less distributed process that extends from the brain to the body and/or the surrounding environment. Extended cognition hence replaces aspects of central processing with more peripheral processing units.

Another important distinction between central and extended cognition relates to their informational requirements. The central cognition conception specifies that specific biological (internal) information is required to process task-specific (environmental) information, and hence that the centralised neural mechanisms, whether they be general or modular, must be sufficiently informed with task-relevant biological information to solve the tasks faced by the organism. In other words, the neural mechanisms must possess or draw on large quantities of biological information^2^ in order to function effectively; they are expected to be informationally greedy. Such biological information concerns or relates to properties of the task, and/or aspects of the world, and hence has a referential property. The most clear-cut examples here would involve communication with signs, whereby a behaviour (a vocalisation, a vibratory pattern) explicitly conveys to others information concerning an object or an event in the external world. Thus, the central cognition hypothesis endorses a representational stance to cognition, in which the external world is, in some way, mimicked or captured isomorphically within the nervous system (Gallistel 1989), for instance, as symbolic representations. Effective behaviour is thought to arise through the individual manipulating these representations, for example, planning future behaviour, prior to performing motor actions. The central cognition conception is informationally greedy because the brain either already possesses (i.e., in a nativist account) or through experience constructs (i.e., in an empiricist account) internal models of the external world. In contrast, extended approaches to cognition are either not representational, or at least require fewer representations, in their conception, and thus are less demanding in terms of the informational requirements of the brain. On this view, informational content is offloaded to the body, or to aspects of the world (Shapiro 2010; Pfeifer et al. 2014; Cappuccio 2016).

Two further points are important here. First, at least in natural systems, information is a fundamentally relational property that pertains to particular organisms, and only exists to the extent that a communication channel is operating that allows the organism to read or extract it. Information cannot exist *solely* in the external environment. Second, while the notion of extended cognition inherently implies a trade-off between information stored in the brain and information distributed beyond it, any such trade-off operates within a species and not necessarily between species. Although it is hard to quantify, we envisage that different species of organisms will vary in the gross quantity of acquired semantic information that they possess, and hence that there is no reason to expect that large-brained organisms will not exhibit extended cognition: to the contrary, humans would seem a prime example.

The idea that cognition extends towards the body, artefacts or the nearby environment holds similarities with the idea of ‘extended phenotypes’, adaptations expressed outside of the organism that constructed them (Dawkins 1999). However, while both concepts deal with artefacts and modified environments, any similarities are superficial. Dawkins characterises all phenotypes, be they extended or not, as the expression of information perceived to be encoded in genes; in this respect, his position is consistent with internalist models of cognition, since the ‘controlling’ information is thought to reside inside the organism. Internalist models are at odds with a view of information as a relational concept, a view that implies that the relevant information emerges only within a system that includes the interaction between the components that constitute the system (among which some external devices could be included). The relational or systemic view of information is embraced by the extended cognition approaches in its embodied, extended, and particularly in its enactivist versions. Extended cognition is more akin to the niche construction perspective (Odling-Smee et al. 2003), because it implies reciprocal causation between the organism and the artefact, or modified environment (Laland 2004). In this way, despite the superficial similarities between the extended cognition and the extended phenotype approaches, they are in opposition when it comes to some of their core assumptions.

Brains are energetically costly organs and, from the allometry between brain and body size (Haller’s rule, Striedter 2005), it follows that the maintenance costs of a large brain, demanded by the central cognition approach, is a particular challenge for tiny animals. These costs mean that smaller animals face more severe trade-offs between energy demands and information processing in their nervous systems (Niven and Farris 2012). Thus, allometry implies that the cost of the brain is proportionally higher for smaller animals, if their small body size is taken into consideration. In other words, it should be harder for smaller than larger animals to maintain energetically costly large brains, but (from the central cognition perspective) reducing brain size potentially leads to a reduced cognitive capacity, and errors in information gathering and processing. Consistent with this reasoning, smaller-brained fishes suffer stronger predation pressure and exhibit reductions in learning and gathering of information about predators (van der Bijl et al. 2015; Kotrschal et al. 2013, 2015), and are restricted to live in less complex habitats (Huber et al. 1997), compared to larger-brained fishes. This trade-off should be particularly strong for predators, because predators tend to be large and mobile so as to track diverse, changeable and spatially distinct prey distributions, and thus require the information processing capability to detect and respond to changes in these distributions (Edmunds et al. 2016a). Consistent with this, in mammals, carnivores have larger brains relative to body size than ungulates (Jerison 1973), and a higher trophic position is connected to larger brains in teleost fishes (Edmunds et al. 2016b). A generalist foraging strategy would also require enhanced information processing capabilities,^3^ because (as Ashby’s Law dictates) a wide niche requires a large number of skills to cope with the diversity of resources, predators, parasites, or competitors, while a narrow niche would impinge less stress over the information processing system. Accordingly, combinations of neural limitations are associated with reduced diet breadth in many unrelated insect taxa (Bernays 2001).

These considerations lead us to expect, other things being equal, that predators, of a relatively small size, with generalist habits, are prime candidates for extended cognition, because they should be under particularly strong selection to reduce their relative brain size but maintain their behavioural richness, and one means by which this could be achieved is through offloading brain processing to the body or to the environment. Spiders, as small, generalist predators (Pekár and Toft 2015), have all the above reasons for living in an informationally overloaded world. These considerations do not mean that other organisms do not extend cognition, but nonetheless do potentially leave this particular order of arthropods of central interest to debates over extended cognition.

The above considerations lead us to expect that natural selection will have favoured cognitive strategies in spiders that allow them to deal with a relatively large amount of information processing without paying the costs of exceptionally large brains, including through extended cognition. Spiders are also interesting because they show taxonomically widespread niche-constructing abilities, using silk for many functions (Krafft and Cookson 2012; Eberhard 2014), most notably in egg sacs and webs. Spiders’ webs are diverse in form, encompassing orbs, cobs, sheets, irregular, and even single-line webs, all of them structures with a long evolutionary history (Hormiga and Griswold 2014). These silk structures have been postulated to act as an extension of the perceptual organs of the spiders, enhancing sensitivity through amplification or attenuation of particular vibrations, and allowing them to detect movements of the substrate some distance away (Naftilan 1999; Masters 1984). The hypothesis of the web as an extended perceptual system, the expectation that web characteristics will have coevolved with spider perceptual systems, and the extensive means by which spiders control and regulate their local environment, most obviously through the use of silk, all combine to leave spiders prime model organisms for extended cognition.

In this paper we first provide a brief overview of the literature on spider cognition, concentrating on new findings and domains relevant to the challenge of distinguishing between the central and the extended conceptions of cognition (see Herberstein 2011; Jackson and Cross 2011 or Hesselberg 2015 for more extensive reviews). Differentiating between these alternative accounts of cognition has historically proven rather difficult (Adams and Aizawa 2001; Shapiro 2010). For this reason, we go on to discuss the ‘mutual manipulability criterion’ (which specifies that two entities that can reciprocally alter the state of each other pertain to one-and-the-same system), a straightforward criterion recently developed explicitly to evaluate claims of extended cognition (Kaplan 2012). Finally, we consider three cases where the mutual manipulability criterion can be deployed alongside empirical evidence concerning spider cognition, and in this manner discuss the support that the experimental data provide to the central and extended models of cognition.

## Spider cognition: what is new?

Nowadays, spiders are far removed from their old depiction as hardwired, instinct-driven animals with few learning capabilities. As this brief section will show, spiders behave as if planning routes in advance, show a sense of numerosity, learn conditional tactics of aggressive mimicry, reverse previous learned associations, and adjust their behaviour to altered conditions in a variety of ways. Indeed, the growing literature on spider cognition has even prompted the creation of an immersive virtual reality system for spiders (Peckmezian and Taylor 2015a), turning them into model organisms for research on learning (Peckmezian and Taylor 2015b).

### Route planning

Araneophagic jumping spiders (*Portia fimbriata*), while foraging on orb weavers, watch the motionless prey from a distance, before engaging in a circuitous path, which often involves losing sight of the spider’s prey, so as to appear near the top of the orb-web. From this position, the jumping spider hangs down from a line until she is able to grab the prey in the centre of its web (Jackson and Cross 2011). This route-planning ability has been experimentally demonstrated (Tarsitano and Jackson 1997; Tarsitano 2006) and involves the categorisation of prey types, combined with the specialised use of working memory (Cross and Jackson 2014).

### Learning

Spiders’ capability for rapid learning is experimentally demonstrated by their learning to avoid dangerous ants and formation of a search image of their prey in just a single encounter (Jackson and Li 2004; Hénaut et al. 2014). Spider learning can be sufficiently strong as to override inborn dietary preferences, as in the case of specialised myrmecophagous (ant- or termite-eating) spiders that will prefer less nutritious prey, such as *Drosophila* flies, when raised on these alternative diets (Pekár and Cárdenas 2015). Web spiders have also been shown to memorise the characteristics of a single captured prey, such as the prey type, size and location (Ades 1988; Rodríguez and Gamboa 2000; Rodríguez and Gloudeman 2011; Rodríguez et al. 2013), and to change web properties as a function of previous prey catches (Nakata 2012; Heiling and Herberstein 1999).

If walking onto an orb-web, araneophagic jumping spiders can learn to use alternative vibratory signals of aggressive mimicry, conditional on the size of the prey. Small, easy to catch, orb weavers are stimulated, through the jumping spider’s vibratory signals, to perform a full attack on a fictitious prey item ensnared in the web. Conversely, more dangerous, larger orb weavers are attracted to the periphery of the orb through alternative vibratory signals that do not stimulate a full attack by the resident spider (Tarsitano et al. 2000). The jumping spiders have been shown to learn to produce the deceptive vibratory signal by trial and error (Jackson and Nelson 2011). That jumping spiders can learn this is not surprising, considering that they can learn associations between vibration and other stimuli (Long et al. 2015) and can reverse previously learned associations between stimuli (Liedtke and Schneider 2014).

Jumping spiders are also able to generalise problem-solving strategies and apply these to new cognitive domains (Cross and Jackson 2015). This ability to perform cross-context generalisations could provide the basis of some findings reporting a sense of numerosity among jumping spiders (Nelson and Jackson 2012). The abstraction needed to generalise between contexts and cognitive domains could be at the base of the abstraction needed for having a sense of numerosity, one that has also been found among web-building spiders (Rodríguez et al. 2015). While jumping spiders do not build webs, they do use silk in a variety of ways, including hunting and kleptoparasitism on other spiders’ webs, as a tether to reach otherwise inaccessible prey, for navigation, and reproduction. Hence, they remain candidates for extended cognition.

### Cognition and performance in small animals

One way to reduce informational demands would be to reduce the computational power of the nervous system, at the cost of performance. Animals with reduced size would show fewer and smaller neurons (Niven and Farris 2012), reducing the resolution of sensory systems and the control over motor systems (Chittka and Niven 2009). Nevertheless, the cognitive feats of spiders (reviewed in Herberstein 2011, and Jackson and Cross 2011) do not consistently suggest any clear reduction in performance with reduced body size. For example adult orb-weaving spiders vary in body mass by 400,000 times (including those near the lower size limit for spiders in general), yet there is no evidence of inferior performance due to very small sizes (Eberhard and Wcislo 2011, 2012). Even the tiniest spiders have much smaller spiderlings that are seemingly as proficient at orb weaving as their adult mothers, when it comes to the precision of performance (Eberhard 2007, 2011). This lack of clear constraints in behavioural performance due to miniaturisation raises the possibility of the occurrence of qualitatively distinct ways to deal with information processing in miniature animals.

## The mutual manipulability criterion

The mutual manipulability criterion (MM—Craver 2007) is the dominant means of assessing ‘constitutive relevance’, that is, of determining whether and when an entity is explanatorily relevant to the behaviour of a larger system. The MM’s popularity may perhaps follow from its intuitive plausibility (Baumgartner and Gebharter 2016). As applied to the problem at hand, one would like to know, for example, if a sensory field arising from the hairs (the trichobothria) on the cuticle of the leg of a spider, or even an external device (like the web), is constitutively relevant to the cognitive system, which is usually considered to be located within the confines of the brain. The MM helps us to understand whether the perceptual field, or the web, is part of a larger system that performs a particular cognitive function. According to the MM, if experimental changes in the external entity (the perceptual field or web) leads to changes in the cognitive system (for example, changes in the attention system) and, reciprocally, changes in the cognitive state of the system, entail changes in the external entity, then this entity can be regarded as a part of the cognitive system, and accordingly one can say that cognition extends to include the external entity (Kaplan 2012).

The reciprocal causation emphasised in the MM account allows for robust experimental tests, both through interventions in the putatively cognitive, external component, and in the central (cognitive) system under inspection. Formally, such interventions must comply with some conditions, so as to demonstrate that a putative component, for example, the spider web, is causally connected to another, for example, the perceptual system of the spider (see Appendix for the formal requirements). It must be clear that the MM account of cognition involves reciprocal causality and, as such, the planned interventions must be performed in both directions (e.g. from the web to the perceptual system and from the perceptual system to the web). Also, the experimental set-up must have standard controls for any alternative intervening variable (see “Appendix”), so as to test causality between the putative system components appropriately. In the following section, we review experiments on spider cognition that comply with these requirements and thus allow for a proper test of the hypothesis of extended cognition.

Other criteria have been proposed to demarcate the boundaries of cognition, such as the ‘proprietary demarcation criteria’^4^ or the ‘bandwidth criterion’.^5^ Nevertheless, the MM criterion outperforms these criteria, because it is better able to distinguish causally relevant components of cognition from background conditions, lower-level correlates, and/or causally inert connections (see below), which constitutes the sole criteria for empirical tests of hypotheses concerning extended cognition (Kaplan 2012).

The proper use of MM circumvents two major criticisms of extended cognition: the ‘coupling-constitution fallacy’ (Adams and Aizawa 2001, 2010), which is the argument that the coupling of an element to a system does not imply that this element is constitutively relevant to the system, and the concern that cognition will be extended to objects only indirectly and distantly connected to the cognitive system through a long and networked causal pathway (Rupert 2004), a possibility labelled ‘cognitive bloat’.^6^

With respect to the coupling-constitution fallacy, we agree that coupling does not inherently entail constitution. For instance, although cognitive activity is coupled to regionally enhanced blood flow in the active brain area, blood flow is not considered to constitute the cognitive process. However, such examples fail the MM criteria: they are coupled to cognition through a one-way, but not through a reciprocal causal path (in this instance, experimentally induced increases in blood flow in a brain area is not expected to lead to increased neural activity in that area). Thus, the reciprocal causality eliminates the possibility of erroneously taking background conditions for components of the cognitive system. As Adams and Aizawa (2010) acknowledge, coupling does not entail constitution, but MM is tailored to identify constitutive relations correctly (Craver 2007; Kaplan 2012).

The correct application of MM also eliminates the possibility of a cognitive bloat. For example, the cognition of one person could be said to be causally connected to the workings of a vast knowledge base like the internet, because changes in the internet content perceived by that person would change her brain’s cognitive states. However, any suggestion that changes in the brain workings of one person will immediately elicit change in the state of an external knowledge database that receives simultaneously the input of billions of people lacks credibility. The MM requirement of reciprocal causality prevents cognitive bloat and rules out many other obviously non-cognitive candidate entities. Likewise in spiders, manipulations of prey type or prey size might well change spider predatory behaviour, but prey should not be taken as a component of the spider cognitive system because they do not satisfy the MM criteria (if one changes the functioning of the neural networks involved in the organisation of the attack behaviour this will not lead to instantaneous changes in either the diversity or the abundance of prey in the environment). In this way, the reciprocal causal pathways in the cognitive bloat type of argument are not effective, and thus the MM again emerges as a powerful criterion to exclude causally inert connections.

## Case 1: Attention extends to web threads

Attention mechanisms are important to filter out irrelevant sensory information, helping spiders to focus on germane areas of the sensory landscape, such as potential prey. Web-building spiders can actively focus attention on a particular web portion. They do that by pulling more strongly the web threads on the more profitable areas of the trap, a behaviour that has been shown to lead to enhanced capture success in these web regions (Nakata 2010).

Enhanced attention to specific web areas can be induced by manipulating thread tension. It is possible to increase the tension of particular web sections experimentally, for example, inducing spiders to build their webs over movable supports; one can change the distances between the supports after the web has been built, thus altering artificially the tension of the horizontal, or alternatively of the vertical threads of the web. In experiments in which researchers artificially tensed the radial threads that led to one web area, the spider increased attention to that area, responding more quickly to stimuli coming from that particular region of the web (Watanabe 2000; Nakata 2010).

Naturally, it is also possible to alter the state of the foraging system in spiders, by simply letting the spiders get hungrier. Hungry spiders will increase web thread tension, so as to respond promptly even to usually less noticeable, and less profitable prey, such as small fruit flies (Watanabe 2000).

Spiders can also learn to focus attention on particular areas of the web. Where researchers have experimentally presented prey items exclusively on the horizontal threads of the web, the spiders have learned to pull these threads more strongly, and thus to respond more quickly to prey offered in the horizontal dimension (Nakata 2013).

Applying the MM to the above experimental results seems straightforward. Experimentally increasing the tension of web threads in a particular area of the web will alter the spider foraging behaviour. The reverse is also true, because one can experimentally induce changes in the internal state of the foraging system (changing the level of satiation of the spider), and this results in changes in the tension of web threads. Thus, to the extent that the above results are experimentally correct, there is reciprocal causation between the web and the foraging system, satisfying the MM criterion. Those cognitive processes associated with spider foraging, particularly the attention system, would appear to extend to the web, mediated by behavioural manipulations of the tension of the radial threads.

The radial threads modulate the resonance and the attenuation of prey vibrations, as well as the velocity of their propagation, and thereby promote signal transmission through the web (Landolfa and Barth 1996). Changes in the tension of threads will differentially affect the nature of web vibrations: while longitudinal waves vary mainly with the composition of the silk, the transmission of transversal waves increases greatly with thread tension (Mortimer et al. 2014). Tense threads increase the amplitude of some and reduce the amplitude of other frequencies, an effect that can also be obtained by increases in thread diameter (Mortimer et al. 2015). This means that the spider can effectively change the transmission properties of the silk, and its processing of vibratory stimuli, by varying its tension. Smaller prey would be less able to produce strong longitudinal waves, but the spider is able to modulate the transmission properties in the web, by tensioning its trap, so as to change her own sensorial input. In this manner, spiders are able to tune the overall attentional system extended through the web to become more sensitive to distinct kinds of stimuli. In this sense, web threads cannot be understood as passive transmitters, or even passive filters of vibratory information. Thread properties are adjustable and thus can process the same information in different and adaptive ways.

The spider regulates thread information processing so as to decide whether to attack a prey in as adaptive a fashion as she regulates the nervous system. For example, she may activate a courtship neural network instead of a prey capture neural network (neural regulation), if the vibration originates from a male walking over the web instead of a prey ensnared in the adhesive threads. Now, the decision-making process that evaluates whether to attack a prey item is initiated before the vibratory information enters the CNS, while it is still being processed outside the spider body in the threads.^7^ Tensed threads will make the spider attentive to small prey, but the same prey will be ignored under a non-tensed threads context. If we accept that the decision whether to attack or engage in courtship is an aspect of cognition (because the spider regulates processing of information within the respective neural networks), then we must equally consider as cognitive the decision to attack a prey. However, this last decision is based on the regulation of web information processing. Moreover, such web regulation is adaptive, because for hungrier spiders small prey are more valuable than for satiated spiders; the latter accordingly focus on larger prey items by relaxing thread tension.

The extension of the foraging cognitive system to silken threads is potentially ubiquitous in spiders. Web tensing is not a rare behaviour in spiders. It is used for prey detection among groups as diverse as Pholcidae (Japyassú and Macagnan 2004), Scytodidae (Japyassu and Machado 2010), Araneidae (Lubin 1980), Theridiidae (Japyassú and Jotta 2005; Garcia and Japyassú 2005), Tetragnathidae (Yoshida 1990), Nephilidae (Japyassú and Viera 2002), or Mygalomorphae (Coyle 1986) and occurs even among web-less spiders that invade other spiders’ webs (Whitehouse 1986). One of the primary, and basal, functions of spider silk is prey detection, a widespread function present in all major spider groups, from Mesothelae, through Mygalomorphae to Araneomorphae, including Haplogynae and Entelegynae (Coddington and Levi 1991; Blackledge et al. 2009). Silk use is a characteristic feature of spiders (Craig 2003), and diversification in silk use (associated with gene duplication and differentiation) is tied to the diversification of spiders (Starrett et al. 2012). Hence, it would seem that the web tension mechanism is a pervasive, and ancient component of the spider attentional system, and is not restricted to web builders or any highly cognitive sub-group of spiders.

Even outside of web-building spiders, among cursorial spiders, the regulation of the transmission and filtering properties of the substrate could also be affected indirectly, through the spider actively choosing particular substrates. For example, many cursorial spiders forage over leaves, or flowers, and it has been shown that *Cupiennius coccineus* select leaves with particularly high vibration transmission properties, such as banana leaves (Barth 2002b). Spiders could thus possibly actively regulate the transmission of vibrations by choosing different substrates for different purposes, such as prey detection, or courtship. Consistent with this, male wolf spiders are found to prefer habitats that transmit better the vibrations (such as leaves) in the courtship context (Gordon and Uetz 2011).

## Case 2: Extended web-building algorithms

We have seen above that radial thread tensing is a component of the attentional system, modulating attention to different web areas and/or prey. The tensing of threads is certainly a small portion of the overall spider foraging system, which comprises much more than attention to prey. Foraging involves many distinct behaviours: the spider has to search for a suitable place for web-building, she has to build and repair the web, detect and capture the prey, and monitor site quality for web relocation (Shear 1986). Among these behaviours, web building is probably the best studied, with a long tradition of natural history descriptions (Tilquin 1942; Savory 1952; Witt et al. 1968), as well as experimental data concerning a variety of influences on web building. This work reveals the spider’s web to be a structure exquisitely dependent on both major environmental features (Scharf et al. 2011) and self-built cues, based on precise interactions between the spider and actual thread configuration (Peters 1970; Ades 1995).

From a functional perspective, web builders modify web dimensions and mesh size in response to exposure to different prey types and sizes (Sandoval 1994; Schneider and Vollrath 1998; Murakami 1983; Heiling and Herberstein 2000). Long-term learning seems important to some of these adaptive structural changes (Heiling and Herberstein 1999, Venner et al. 2000).

Descriptive and experimental work has been performed to understand web-building rules (Eberhard 1972, 1990; Zschokke and Vollrath 1995; Zschokke 1996, 2000, 2011). This research suggests that spiders use many distinct external cues while building the web, such as prey-induced vibratory stimuli and prey nutrients (Pasquet et al. 1994; Blamires et al. 2011), wind intensity (Wu et al. 2013), gravity (Witt et al. 1976; Eberhard 1987; Vollrath 1988a, b), and humidity (Baba et al. 2014). Spiders also use internal cues to guide web building, such as the amount of silk supply, spider size, weight (Eberhard 1988a), and leg length (Witt et al. 1968; Vollrath 1987).

A spider also relies on cues that she herself has produced, through building earlier stages of the web, using the configuration of previous laid threads to organise the next steps of web building (Peters 1970). These cues include the spider position in the web (near or far from, and above or below, the hub), or cues from sticky lines already present in the web that are sensed anew on each radius (the site where the preceding loop of sticky line was attached to the radius, and the distance from the outer loop of temporary spiral to this inner loop of sticky spiral—Eberhard 1972, 1982, 2012a). Moreover, while walking on previously laid lines, spiders do not solely respond automatically to present stimuli, but also rely on memory and attention. Spiders also do not invariably respond instantaneously to the immediate thread configuration because in many contexts spiders walk long journeys (relative to their body sizes) from one point of the web to another, and must keep track of the distances travelled in order to fix the new line at a precise point: they need to memorise the distances travelled at each step, while building the trap (Eberhard 1988b). Frequently the relevant cues are not immediately available (Eberhard 2012a, b), so that the spider must decide where to fix its current thread based on memories about previous decisions in the web-building process. Memory and attention are integral parts of web building, and it is probable that these cognitive processes are even more relevant in the initial, exploratory phase of web building (Hesselberg 2015).

**Fig. 1:**
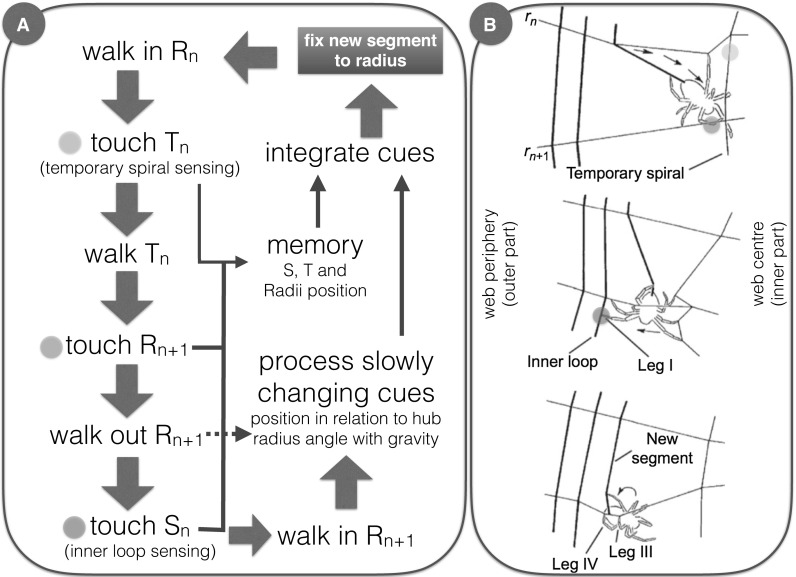
Cycle of actions necessary to build the current segment of the adhesive spiral. Steps and processes within the cycle (**a**), with the illustration of some of the behaviours involved (**b** adapted from Eberhard and Wcislo 2011). The cycle (*blue arrows* in **a**) begins and ends with the fixation of the current adhesive spiral segment (*blue box*). The spider fix (the current segment) over the current radius (R_n_, **a**; spider behaviour displayed at **b**, *top figure*), and then in the next radius (R_n+1_, **a**; spider behaviour displayed at **b**, *bottom figure*). The spider performs successive actions (*large blue arrows*, **a**), while assessing the position of some rapidly changing cues (*coloured balls*, A and B). Slowly and rapidly changing cues are stored, compared to each other (to obtain distances and rates of change) and then integrated (*continuous* and *thin blue lines*, **a**) to determine the position of the next adhesive segment fixation (in R_n+1_). When confronted with conflicting cues, the spider may ignore some cues (inner-loop sensing), and proceed to a shortcut, an alternative routine (*dotted blue line*, **a**). These cycles are repeated until the completion of the capture area

One example of the complexities involved in web-building is the decision about the distance between successive sticky spiral segments in one specific radius (Fig. 1). This decision is repeated at each new attachment point and involves the assessment of many distinct cues, such as reference points (the position of the inner loop of sticky spiral; the position of the outer loop of temporary spiral), the distance from the hub, the angle of the radius with gravity, the distance between radii, the measurement of distances (such as the actual temporary spiral to inner-loop distance) and the comparison of these distances with either short-term memories of similar distances in the previous sticky spiral segment attachment, or less recent memories regarding the attachment of the previous sticky spiral loop, in the same radius, among others (see review at Eberhard and Wcislo 2011; Eberhard in prep). Due to the complexity of the task, sometimes spiders ignore some cues in favour of others. For example, while building the sticky spiral spiders can be faced with conflict between distinct cues; removal of the temporary spiral in one sector of the web (which can be done experimentally, or by the spider herself) introduces conflict between the actual and the previous temporary spiral to inner-loop distances, and in this case the spider frequently ignores one set of cues (for example, inner-loop sensing) in favour of others (previous temporary spiral to inner loop, Eberhard and Hesselberg 2012). The reverse effect also happens in natural or experimental webs, when there is an over-sized distance between the preceding sticky spiral loops: in this situation the spider sometimes ignores the inner loop cue in the actual radius and overshoots the actual inner-loop segment attachment point, thus producing a reduced distance between the actual and the previous sticky spiral loop, so as to compensate for the over-sized distance between the preceding loops (Eberhard 2011, in prep). Thus, by manipulating the actual configuration of threads during the web-building process, the experimenter can actively change the spider’s attention, leading the spider more prone to ignore some cues in favour of others.

The spider’s use of memory can also be deduced from more long-term and global changes in web structure that result from learning. Spiders will increase the size of a particularly profitable web area, leading to changes in web symmetry, if offered prey preferentially in the region below the hub (Nakata 2012; Heiling and Herberstein 1999). Similarly, spiders detecting prey without capturing it increased the total thread length and capture areas of their webs compared to a control group (Nakata 2007). Evidence of learning also stems from forcing spiders to build in abnormal conditions. *Argiope argentata* usually builds vertical orbs; if forced to build in horizontal cages, her first web is very irregular, but subsequent ones become progressively similar to regular orbs (Nogueira and Ades 2012).

Manipulations of regions of the CNS involved in web building have also been performed. Spiders with laser induced lesions in the C3 region of the supraoesophageal ganglion build smaller and rounded webs, with reduced regularity in the positioning of repetitive components (Witt 1969). Interventions in the CNS with neurotoxins also resulted in significant changes in web properties. Chlorpromazine, diazepam and psilocybin prevent onset of web building and sodium pentobarbital causes end of radius construction before completion. D-amphetamine causes irregular radius and spiral spacing, while LSD-25 results in unusually regular webs (review: Witt 1971). Amphetamine, scopolamine and caffeine cause various alterations in web geometry (Hesselberg and Vollrath 2004). Substances present in potential prey (gonyleptidine), such as harvestman, cause the construction of more irregular webs (Albín et al. 2014), and some parasites even manipulate the web-building behaviour of their host spiders, injecting substances that cause them to build altered web structures to be exploited by the parasites themselves (Eberhard 2000a, b; Matsumoto 2009). The effects of these substances on web structure reveal the targeting by parasitoids of areas of the CNS that regulate web-building rules (Eberhard 2010). It seems abundantly clear that changes in the functioning of the CNS, particularly in some specific areas of the supraoesophageal ganglion, have substantial effects on the webs built by the spider.

In spiders, hunger levels also have clear effects on foraging behaviour, particularly web building. Well-fed spiders build orbs less frequently (Vollrath and Samu 1997), with smaller capture webs (Mayntz et al. 2009; Baba and Miyashita 2006, but see Vollrath and Samu 1997), and also webs with an added structure, the barrier web (Baba and Miyashita 2006). This result is not restricted to orb weavers: well-fed cob weavers also decrease the amount of silk used in the capture area (viscid silk, number of anchor lines, and sheet of the web), while increasing the amount of silk used for supporting or barrier structures (Blackledge and Zevenbergen 2007). Hunger levels change foraging behaviour not only in web builders, but also in other trap builders (Scharf et al. 2011), and even in wandering spiders (Okuyama 2011; Aguilar-Argüello and García-Chávez 2015).

From the literature reviewed above, it becomes clear that one can alter the attentional state of the spider to web-building cues by changing thread configurations. This is an intervention on the web threads promoting a change in cognition. The reverse intervention also reveals connectivity between web structure and its underlying cognitive system: the manipulation of the functioning of parts of the CNS resulting in web-induced changes is abundantly documented and, more generally, the effects of specific neural networks on foraging activity are also well documented, for hunger regulates the activation of motor activities in invertebrates through 5-HT neurons embedded in the feeding motor network, configuring a promoter of appetitive state (Gillette 2006). The evidence is thus that the MM can be applied to the foraging system, more specifically to web building, implying that the web-building cognitive machinery extends from the spider brain to the web itself.

The claim of the extended cognition approach would be, in the case of web building, that the use of the structural connections and organisation of the web as integral components of the cognitive system itself would reduce the necessity for cognitive processing within the CNS. This could be achieved, for example, by a reduction in the need for long-term spatial memory. It is well known that some spatial tasks require long-term spatial memory and that the performance of these tasks is associated with increases in brain size. For example, many birds cache food for later retrieval (Balda and Kamil 1989; Shettleworth 1990) and cannot rely on any clear-cut environmental cue for retrieval, because otherwise conspecifics would readily pilfer these easy caches (see review by Keefner 2016). In this way, birds have to memorise the location of literally thousands of caches (Pravosudov and Roth 2013), and this enhanced reliance on spatial memory is correlated with larger hippocampal volumes and neural numbers, either across populations (Roth and Pravosudov 2009; Croston et al. 2015) or across time (within individual seasonal variation, Smulders et al. 1995, 2000).

Birds cannot solve their food-retrieval problem without long-term spatial memory, but spiders could reduce the usage of long-term memory and still solve the spatially challenging problem of building a web. To build each orb-web, spiders make thousands of measurements, keeping track of the distances travelled so as to make decisions throughout the process. But while birds cannot rely on the stability of the external environment for cueing food retrieval, spiders can rely on previously laid threads as cues for new navigation decisions. Since web threads are reliably out there while the spider is building its trap, there is no need to memorise all the details of the emerging structure (each of the distances travelled between spiral segments, radii distances, the relative position of the spider at different moments), because at each new step of the building process the spider can reset the memory used in the previous step. For example, at each new fixation of one spiral segment, the spider can forget the distance memorised for the fixation of the previous spiral segment. Thus, the spider is able to trade long-term for short-term spatial memory, simply because the threads already fixed will remain in place, cueing the next steps. Plausibly, natural selection may have favoured extended cognition here because it allows the use of operational memory, whereas centralised cognition would require more intensively long-term memories, to solve the task. After operational memory is erased, the CNS circuitry responsible for this memory can potentially be available for the memorisation connected to the next behavioural routine (i.e., in a sense, operational memory is renewable). A simple thought experiment helps to make clear the substantial economy in cognitive resources provided by the silken lines already in place: imagine a spider that performs exactly the same movements that a regular spider performs to build a web, only that this imaginary spider does not lay down silk, but rather ‘builds’ a web without leaving traces of her path. To do this, the imaginary spider would have to memorise the whole path, and all the decisions previously taken, so that she would not pass two times on the same place, or fix ‘spiral segments’ twice in the same point of the same ‘radius’. This is the kind of memory that a real spider does not need, because she uses previous threads as physical memories of her path.

This is not to suggest that web building does not need long-term memory: it does. Rather, the point here is that the spider, by relying on the previous threads as external, long-term memory devices, probably requires less CNS long-term memory than other similarly complex animal activities (that must instead rely mostly on centralised cognition). Spiders are not alone in using external cues to simplify complex navigation tasks (see review in Shettleworth 2010), but they are certainly an outstanding example because, since they build their own externalised memory traces, these built ‘environmental’ cues are extremely reliable, allowing the cognitive system to evolve in the direction of extending itself to encompass the previously external, niche-constructed environment (the web itself). The same logic applies to any niche-constructed device (burrows, retreats) built by one single individual, and thus extended cognition could also play a part in the evolution of these structures.

The reliable permanence of threads already laid by the spider, while web building proceeds, can also possibly reduce the computations needed to finish the structure. For example, instead of calculating in advance the number of radii needed to complete the web, the spider can proceed by filling in open spaces with new radii, until there is no open space left. By the same reasoning spiders would not need to calculate the number of viscid spiral loops before actually laying these threads, because the emerging structure itself helps to simplify the task, reducing the need for complex geometric calculations. In a way, we could say that threads simplify the problems faced by this almost blind animal by reducing the dimensionality of the navigational problem. Instead of navigating on a fully three-dimensional space, spiders basically navigate through one-dimensional draglines (even cursorial spiders leave silken lines while walking). Silken lines could shrink the effective dimension of the space that spiders navigate through, helping thus to reduce the complexity of the task. For example, when an orb-weaver is disturbed she usually jumps out of the web and hides among dead leaves in the forest litter, but later returns to the web. Finding the way back to the web could be a challenging navigational problem for a blind animal, but the task is trivial for the spider, because all she needs to do is to climb back through the safety dragline she left fixed at the hub right before she jumped away from the web. In these ways, extending cognition to the web would not only outsource information processing, but would also reduce the overall need for information, simplifying the immense navigational problem that a tiny blind animal would face so as to end up with an optimal geometrical solution to a fundamental foraging problem.

## Case 3: Matched filters do not extend cognition

From the previous examples, we have been able to conclude that some external components (web features) are integral parts of the spider cognitive machinery. We now move on to show briefly how the application of the MM criteria can also rule out potential candidates for extended cognition.

**Fig. 2:**
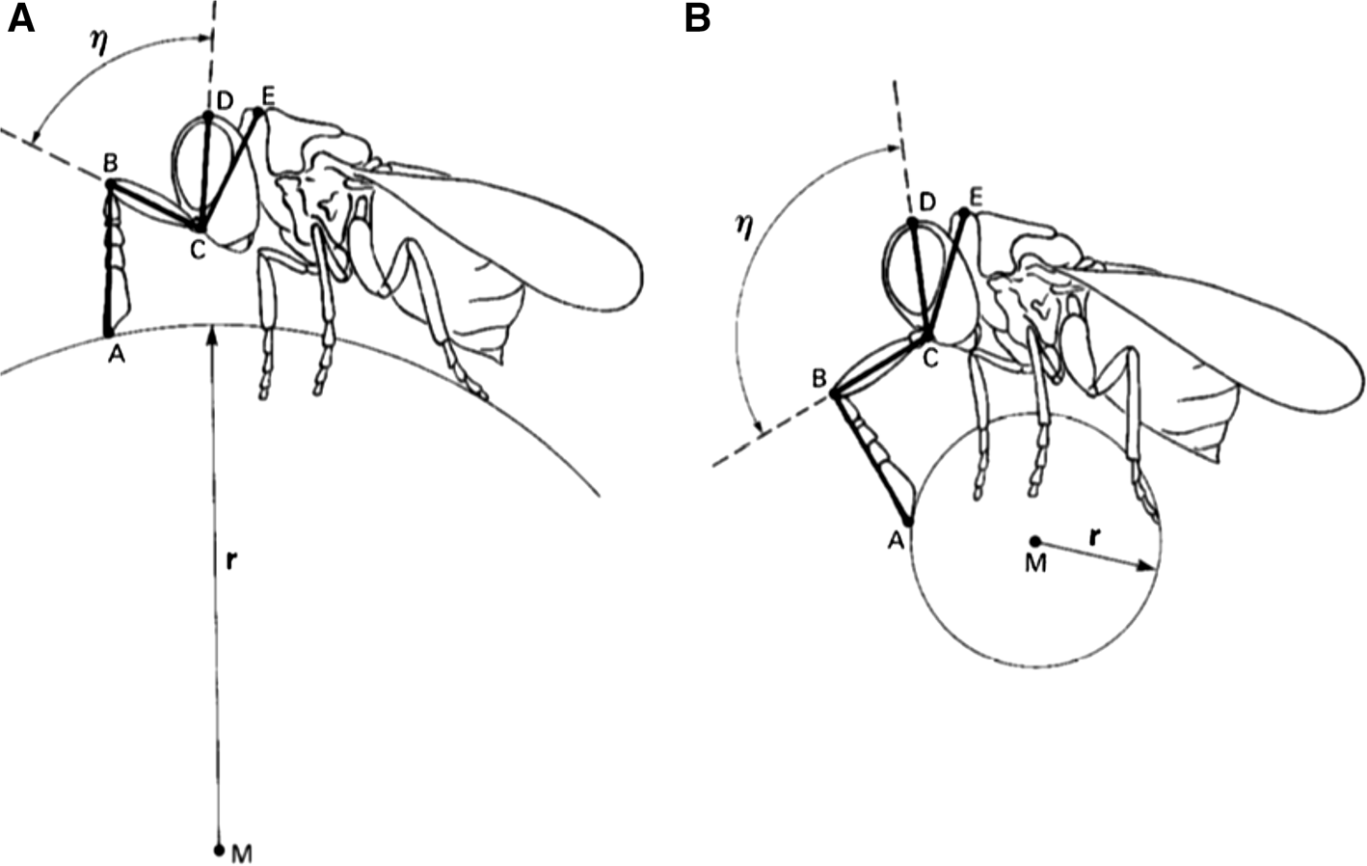
Matched filters provide high-level information to the CNS, but do not extend cognition. Parasitoid wasps use the curvature (angle BCD) of the host insect egg to determine the number of progeny allocated to it. The angle between head and scapus (angle BCD) is correlated with the radius of the egg. Figure adapted from Wehner (1987)

Matched filters are sensory systems that are tuned to specific aspects of the sensory landscape, so as to resonate with that particular aspect, filtering out irrelevant stimuli. A classical example would be that of parasitoid wasps of the genus *Trichogramma* (Ichneumonoidea), that match the number of their own progeny to the volume of each particular host egg (Schmidt and Smith 1986). The trick can be easily solved at the sensory level. The wasp determines the radius, and thus the volume, of her host (egg) not by performing some kind of spherical trigonometry, but by taking a single measure—the [wasp’s] head/scapus angle—monitored most probably by the mechanosensory bristles located at the joint between head and scapus, when the wasp is walking over the host’s egg (Fig. 2). Thus, particular receptor distributions could incorporate fundamental aspects of the ecological problem, providing straightforward, high-level information to simplify otherwise relatively complex tasks (Wehner 1987).

Spiders have many different kinds of matched filters. The organisation of the slit sensilla (Blickhan and Barth 1985) and lyriform organs (Barth 2002c) adequately map the stress lines produced in the leg cuticle by normal stimulation. Prey walking onto a surface produces a high-frequency, broad-band spectrum of vibrations, which are very distinct from the low-frequency, narrow-band spectrum noise produced by abiotic factors such as the wind. The slits of the metatarsal vibration detector are high-pass filters, relatively insensitive to low frequencies (Barth 1982), and the primary sensory cells block the low frequencies typical of background noise (Barth 1998), thus filtering out unimportant stimuli from the sensory landscape.

Trichobothria (hairs in the cuticle, deflected by air flow) can have different lengths, and the longer they are, the lower the frequencies at which they resonate (Barth et al. 1993; Humphrey et al. 2001). Groups of trichobothria with slightly different sizes represent a set of bandpass filters that enhance sensitivity. The air-flow fields generated by abiotic factors, for example, in tropical forests, are characterised by low-frequency and narrow-band spectrum, with low velocities, while the air-flow fields produced by prey items, such as flies, are characterised by high frequencies and wind velocity. Since the proportion of high frequencies in the spectrum increases quickly as the fly gets closer to the cursorial spider, the set of bandpass trichobothria hairs can process directly the information about the distance of the prey, enabling a quick and precise lunge. Spiders with ablated trichobothria do not jump towards flying prey (Barth 2002a).

Although it is clear that matched filters process biologically relevant information coming from the environment, as ablation experiments show, there is no indication that changes in the central nervous system of spiders will lead to changes in the functioning of the sensory organs themselves, a requirement of the reciprocal causation embedded in the MM. As the cognitive system of spiders most certainly cannot actively alter the functioning of the sensory organs, cognition does not extend to the matched filters, according to MM.^8^ While matched filters do not satisfy our criteria for extended cognition, they may, nonetheless, be examples of embodied cognition, which is a broader related concept.

## Advantages of extended minds

Extended spider cognition may help explain some rather unexpected experimental findings. For example, if web building is conceived as a centralised cognitive process, researchers might reasonably expect significant amounts of brain tissue to be dedicated to the organisation of the various behavioural units needed to obtain the final web. On this view, the ability to build a complex structure such as an orb-web should be correlated with those brain areas devoted to the processing of the multitude of simultaneous cues, of the different kinds of silks and spinnerets to use in different situations, of the organisation of the various components of the structure, such as frame lines, temporary spiral lines, radii, viscid spiral lines, hub building and rebuilding, while monitoring the spatial position of the spider within the emerging structure, and the properties of relevant environmental features, such as gravity, humidity, wind, prey abundance and type, etc. From the centralised cognition standpoint, these aspects of the web should be represented internally, in the organisation of a potentially relatively large and costly CNS tissue that would yield high foraging payoffs.

Conversely, from the extended cognition perspective, if the cognition for web building extends to the web itself, the amount of CNS neural tissue dedicated to web building would be expected to be significantly smaller than what the centralised cognition stance anticipates. It follows that the two approaches to cognition have contrasting predictions about the relative size of spider CNS tissue dedicated to solving specific tasks. Now, within the group of orb weavers, web building has been lost or simplified independently several times. For our purposes, the loss of web building among the kleptoparasitic genus *Argyrodes* is particularly relevant, because these spiders are particularly small in size, and smaller sizes involve higher maintenance costs for relatively larger brains (Haller rule, Striedter 2005). Miniaturisation should be accompanied by increased pressures for reducing relative brain size, because of a trade-off between energy demands and information processing in their nervous systems (Niven and Farris 2012). Indeed, from the centralised cognition standpoint, the loss of web building in *Argyrodes* could be thought of as an evolutionary response to these pressures, allowing these spiders to dispose of relatively large brain areas devoted to web building. Nevertheless, notwithstanding the loss of web-building ability, these tiny spiders did not change their relative brain size (Quesada et al. 2011). This result is unexpected from within the usual central approach to cognition, but is precisely what an extended approach to cognition would predict.

**Fig. 3:**
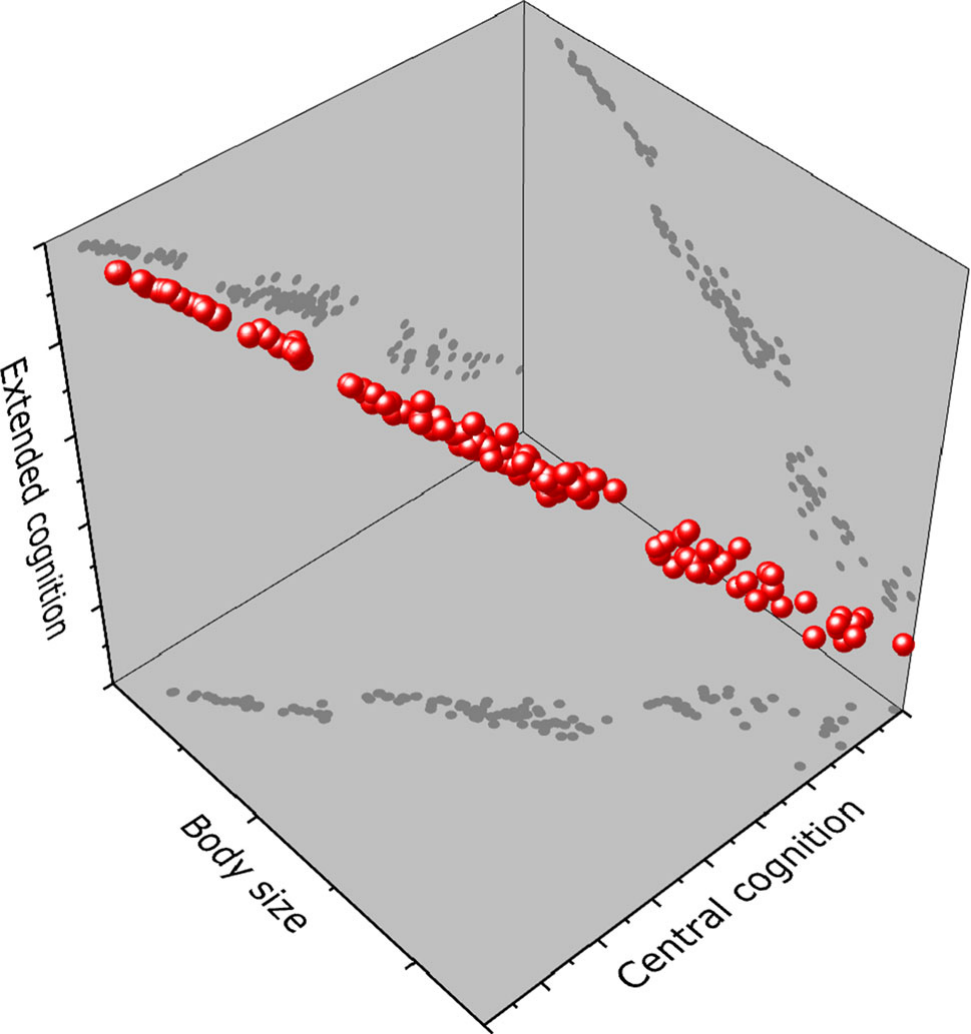
Graded changes in brain–body allometry (*grey dots* at the basal plane, positive Central cognition-body size correlation) do not follow from Haller’s rule. A possible explanation for the stepped correlation across taxonomic groups (each of the three distinct clouds of points) would be the relative amount of non-brain based information processing (negative correlation between Extended cognition and Body size, *grey dots* at the right plane). Relatively higher degrees of extended cognition would help animals to maintain performance at much smaller body sizes. The correlation between cognition (both central and extended) and body size is represented by the *red dots*

Extended cognition could also help to explain an unsolved puzzle in comparative analyses of brain–body allometry. It is well known that different taxa have different slopes and elevations of brain–body scaling (Fig. 3). Larger animals such as mammals or birds cannot be as small as ants, because they would have prohibitively large brains (Eberhard and Wcislo 2011). This shows that small-sized animal taxa have apparently solved scaling problems seemingly insuperable for larger animals (Eberhard and Wcislo 2012). One possible explanation for these brain-scaling taxonomic differences would be that these tiny brains harbour a simpler behavioural system, but this does not seem to be generally the case, when one looks, for example, at the cognitive feats of jumping spiders (reviewed above), or bees, that seem capable of complex learning, including the formation of concepts (Giurfa et al. 2001; Leadbeater and Chittka 2007), or even tiny spiders that do not have reduced ability to build functional orbs (Eberhard and Wcislo 2011; Hesselberg 2010). Another possible explanation would be at hand if these tiny brains were inherently less precise in their performance, but that also does not always seem to be the case (Eberhard 2011).

Alternatively, tiny brains could be more richly modulated than larger brains. Neuromodulation could help the nervous system to multitask, by multiplexing the network (Wang 2010; Akam and Kullmann 2014). Although the available information about neuromodulation in spiders does not suggest that spiders have particularly sophisticated neuromodulatory systems (Seyfarth et al. 1993; Widmer et al. 2005; Jones et al. 2011; Torkkeli et al. 2011; Barth 2013; Hebets et al. 2015), the dynamic properties of spider nervous system networks have just begun to be studied with new neurophysiological techniques (Menda et al. 2014), so that this possibility can now be better investigated in live animals.

One final solution to this brain–body scaling problem would be the outsourcing of processing power from the centre to the periphery of the nervous system/body, or to organised and constructed areas of the external environment: that is, to extend cognition. If these smaller animals find ways to regulate the properties of features of their environment through their behaviour (as spiders alter the properties of web threads by increasing tension), or if the organisation of these environmental features help to assemble together different behavioural domains (like in the case of building silken sheets, see below), the stress over centralised control is reduced, allowing a tiny brain to perform better (Fig. 3).

Miniaturisation is a common phenomenon and is perhaps becoming yet more common as the increasing temperatures in the Anthropocene select for smaller body sizes (Sheridan and Bickford 2011). Miniaturisation is consistently correlated with morphological simplification and novelty (Hanken and Wake 1993). For behavioural systems, simplification is often understood as connected to reduced behavioural plasticity, because simpler systems would have lower number of internal stable states, hence a lower number of behavioural outputs (Arnellos and Moreno 2015). Considering the web-building system, spiders have repeatedly evolved from orb to sheet or cob weavers (for example in Linyphiidae, Theridiidae, and Nesticidae: Bond et al. 2014), and this change is connected to major simplifications, with the loss of whole phases of the ancestral orb-web building algorithm (Benjamin and Zschokke 2002, 2004). Nevertheless, instead of exhibiting less plastic performance, these derived species of spiders are even capable of larger adjustments in web design relative to ancestral forms. The derived forms have typically lost the stereotypic construction behaviour (Benjamin and Zschokke 2003, 2004) and gained ample intra-individual, inter-individual, and species variability (Eberhard et al. 2008). This complexity of behavioural outputs (i.e., web variability), which arises in spite of apparent simplicity in underlying behavioural rules (e.g., a web algorithm), requires explanation. Extensive web variability and innovation appear connected to a higher behavioural imprecision in these hyper-variable spider groups (Eberhard 2000a, b; Eberhard et al. 2008), which could be obtained, at a mechanistic level, from the simultaneous loss of several cues for setting various web parameters, particularly the loss of an important organiser of orb-webs, the delimiting frame threads (see Eberhard et al. 2008). The absence of frame threads, of radial organisation, and of the whole phase of temporary spiral building turned the cob and sheet web algorithms much more dependent on the initial, exploratory phase of web building (Benjamin and Zschokke 2004), a phase that is much more variable, mainly involving the fixation of threads over the substrate (Hesselberg 2015). The dependence of the exploration phase (and independence of the remaining phases) on the substrate has been experimentally demonstrated for the ancestral orb weavers: while the length of the exploratory phase increases with the complexity of the substrate, the length of the remaining phases does not match substrate complexity (Zschokke 1996). Since these remaining phases are either absent or much less organised in derived cob or sheet weavers, the net result is a web much more dependent on the exploratory phase, and thus much more connected to the substrate. As a result, these webs seem to incorporate substrate organisation into the organisation of the web itself, and in this way substrate variability could help explain the large intra-individual, inter-individual and species variability in the group. What we see is a decrease in the dependence of central organisers (simplification of web building algorithm) with a simultaneous increase in the dependence of external organisers (substrate form), resulting not only in more plasticity, but also in the evolution of a huge diversity of web patterns (Eberhard et al. 2008). Thus, the outsourcing of information from behavioural algorithms to the environment could not only help to explain the huge success of a diverse group of spiders, but could also help to explain the taxonomic diversity of this group.

Extended approaches to cognition could also shed light on some other aspects of spider behaviour. Beside webs, spiders build a variety of silken structures, such as barrier webs, retreats and stabilimenta. These structures involve the filling of a surface, either over a substrate, or suspended in the air, which is obtained with the repetition of behavioural routines of fixing threads over threads while walking over the emerging structure. These silken surfaces have evolved multiple times within distinct, independent spider groups, and they have also appeared many times independently among insects (Craig 1997), or pseudoscorpions (Tizo-Pedroso and Del-Claro 2005), whenever silken lines evolve in the first place. The production of a silken sheet thus seems straightforward, in the presence of the ability to produce silken lines. One possible explanation for the ease with which evolution finds behavioural algorithms for building silken sheets is that it is also common for animals to have territories, areas that are well known and explored intensely, and that are frequently marked for recognition, chemically or otherwise. If an animal is able to produce silk, territory marking with silk is straightforward, and if walking within a territory is common, nodal areas in the territory (central resting places) will be filled with a tissue of silk (Edgerly et al. 2002). The production of silken sheets could be organised through positive feedback loops between behaviour and silk lines: if the animal marks its path with some volatile chemical compound in the silk, the most walked through places will retain the most of the marking, and the attraction to the denser regions of frozen paths would feedback into the system, leading to the emergence of silken tissues. Thus, the conjunction of territoriality with the ability to produce threads could result in the production of silken sheets, not because of some cognitive faculty that organises behaviour from within the CNS (central cognition), but instead because of the self-assembly between distinct aspects of the behaviour of the species, promoted by enduring niche-constructed aspects of the environment. In this way, a dynamic systems approach to cognition could help explain the emergence of less CNS-demanding, but still complex, behaviours. Given this easy first start, the evolution of silken sheets for particular purposes (protection, prey capture, courtship) would require only a few complementary organisational steps (which could even be based on central cognition).

Although self-reinforcing loops of this kind are well known to produce organised output in many distinct dynamical, including social, systems (Couzin 2009; Sumpter 2010), it is important to notice that extended processes always work in conjunction with centralised organisers, which allows the extended components to evolve. Extended cognition cannot entirely replace centralised cognition; otherwise, the autonomy of the living system would disappear in a constant flux of ecological relations. Within social systems, extended cognition would be a logical intermediary step facilitating major evolutionary transitions (sensu Szathmáry and Maynard-Smith 1995), a step that nevertheless further requires means to circumvent conflicts of interest within the newly emerging social system.

## Conclusions

The literature we have reviewed, allied to the mutual manipulability criterion, leads us to conclude that spider cognition does extend to web threads and its configurations, but not to matched (sensorial) filters. Although matched filters process information adaptively, the cognitive system must simply take this information as input for its workings, and cannot manipulate directly the state of these sensory organs. The mutual manipulability criterion is thus very discriminating and allows researchers to detect the boundaries of the cognitive system effectively.

Despite allowing a straightforward distinction between centralised and extended cognition, the MM is also experimentally demanding, requiring interventions on the CNS and at the periphery of the nervous system (or in the niche-constructed environment). As a consequence, some well-known examples of spider cognition, like route planing and other impressive cognitive feats of jumping spiders, still require further investigation to allow the application of the MM criteria. Accordingly, we believe there are good opportunities for further experimentation leading to the recognition of other aspects of spider behaviour as extended cognition.

More generally, spiders and other small-sized invertebrates (and perhaps even many small vertebrates) are natural candidates for the discovery of further extended cognitive mechanisms, because these extended mechanisms would be especially relevant for small-brained animals. Small-sized animals may have solved the brain–body scaling problems posed by miniaturisation by outsourcing information processing, that is, by extending cognition to the most peripheral parts of their bodies, or to the closest elements of their environment. This ingenious solution may be particularly successful when this closest environmental feature is produced by the organism itself, as in the case of nests, burrows, webs, retreats, and other artefacts produced by animals, because coevolutionary loops could then fine tune the properties and use of these artefacts more closely, allowing cognition to extend in a more complete form. It may be no coincidence that some of the most cognitively sophisticated invertebrates (e.g., social bees, wasps, ants) are renowned for their niche construction (e.g., nest building). We thus have a double prediction: that miniaturisation will select for extended cognition and that niche construction will facilitate the process of outsourcing information processing.

Moreover, by outsourcing cognition and building structures, organisms potentially make their world more predictable, and a predictable feature of the environment will frequently turn out to be a selective pressure (Odling-Smee et al. 2003). As a consequence of having a web, selection pressures appear for the web to be defended, maintained, regulated, and improved upon in design, as well as for others to hunt on it, destroy it, squat in it, or steal food from it. Such adaptive responses potentially evolved multiple times, perhaps producing parallel evolution in independent lineages, and/or long-term evolutionary trends spanning multiple characters, in ways that are potentially probabilistically predictable (Laland 2015; Laland et al. 2015). Such parallel evolution or trends, together with the aforementioned predictions concerning miniaturisation and simplification, could be investigated using comparative phylogenetic methods or through comparative experiments. Hence, increasing attention to the possibility of extended cognition may open up exciting new opportunities for novel research.

## Appendix: Formal requirements to the mutual manipulability criteria

Formally, the interventions realised to detect whether two elements belong to one-and-the-same system must comply with some conditions, so as to demonstrate that a putative component X (for example, the spider web) is causally connected to Y (for example, the perceptual system of the spider): A necessary and sufficient condition for X to be a (type‐level) contributing cause of Y with respect to variable set V is that (i) there be a directed path from X to Y such that each link in this path is a direct causal relationship; that is, a set of variables Z1 … Zn such that X is a direct cause of Z1, which is in turn a direct cause of Z2, which is a direct cause of … Zn, which is a direct cause of Y, and that (ii) there be some intervention on X that will change Y when all other variables in V that are not on this path are fixed at some value (Woodward 2003, p.59). Also, there are some necessary conditions for an intervention (I) to appropriately test the causal relationship between X and Y (Woodward 2003, p. 98):

1. I causes X.
2. I acts as a switch for all the other variables that cause X. That is, certain values of I are such that when I attains those values, X ceases to depend on the values of other variables that cause X and instead depends only on the value taken by I.
3. Any directed path from I to Y goes through X. That is, I does not directly cause Y and is not a cause of any causes of Y that are distinct from X except, of course, for those causes of Y, if any, that are built into the I‐X‐Y connection itself.
4. I is (statistically) independent of any variable Z that causes Y and that is on a directed path that does not go through X.

These are indeed general conditions to test causality in any experimental setting. It must be clear that the MM account of cognition involves reciprocal causality and, as such, the planned interventions must be performed on both directions, from X to Y and from Y to X.

Baumgartner and Gebharter (2016) suggest that MM detects constitutive relevance, but that this does not imply the existence of causal relationships within the extended system, i.e., that constitutiveness does not entail causality.^9^ Nevertheless, this is only true when you consider the relationships between the overall system and its constituent parts, because these are spatiotemporally overlapping relations, that is, the system does not causally act over its own parts, because it is merely constituted by these parts. Of course, this reasoning also works the other way round: the parts do not causally act over the system as a whole, because this would entail a causal loop whereby the part acts over itself. So, this reasoning is tailored to evaluate the relationship between the whole and its parts. Nevertheless, this is not the question we are trying to evaluate. We are not interested in the relationship between the whole extended (cognitive) system and one of its parts (threads, webs, sensorial fields). Instead, we are interested in the relationship between different parts of the system, and thus we do not analyse the system simultaneously at two levels of organisation (at the level of the whole, and at the level of the parts). We stick to one level of analysis, and we analyse only the parts and the interrelations between them: we are dealing with the causal structure among the parts (of the putative extended system), and this is sufficient for our purposes.

Also, the argument just make sense if one considers the macro- and micro-levels not as alternative descriptions of one system, but instead as distinct entities that could be causally independent from each other. This interpretation comes from the correct and well established idea that the whole is more than the sum of its parts, so that the description of the macro-system is more than the description of its parts. But if we consider not only the parts, but also their interactions (additive or non-additive), which are not strictly predictable in complex systems, then the description of the macro- and micro-levels do coincide, and emergent properties will appear within these complex interactions. If a property of an entity change as a function of its interactions (as in multiplexing neural networks, that are differentially activated for different functions—see Wang 2010), then the interactions must necessarily be part of the description of the system, at any (macro or micro) level.

It is not the place here to develop a functional analysis of the cognitive system, but we would like to show briefly that such an analysis would even solve the problem that Baumgartner and Gebharter (2016) remark, namely, that the macro-level is constituted by (and supervenes on) the micro-level, and thus does not causally interact with it. To perform such an analysis we will build on the theoretical development of Moreno and Mossio (2015). These authors show that the macro-description supervenes on the configuration of the parts, while the configuration itself remains as an emergent property of the parts. From this position, there are two relevant points concerning the extended cognition: the web (or any other biological component external to the CNS) must be a constraint for the internal workings of the CNS cognitive system, and it must perform closure (see below) in this very system. As to the first point, to be a constraint, is to channel the functioning of a system (in a cognitive system, to channel information processing in a specific, biologically relevant way), without being changed in the timeframe of the ongoing process, so as to accelerate or to make easier a process. As to the second point, for a constraint to be part of a closed configuration, causal relations between the constraints (internal and external) must take the form of a closed network of interactions. Web threads clearly constrain information processing in the spider’s CNS, and the web and threads as constraints are clearly entangled in multiple reciprocal causal relations to the CNS cognitive machinery (in a technical sense, they perform closure of constraints), as shown in the empirical data we review in this contribution. Thus, an analysis from within this systemic perspective is entirely compatible with the analysis we develop in the present contribution, and would render philosophically sound the description of the system in distinct (macro and micro) levels, that would correspond to distinct causal regimes.

Anyway, for our purposes, we can bypass this discussion, because it suffices to consider the relationship between the macro-level description of the internal cognitive system (the brain, or specific parts of it), and a putatively external variable (the sensory field, or the spider web), without reference to the internal workings of the CNS. Our aim is to evaluate whether the putatively external variable changes the functioning of the CNS and, vice versa, whether changes in the state of the CNS lead to changes in the putatively external variable. It does not matter if the effect of the external variable on the macro-system (and vice versa) occurs through some molar property of the macro-system or through some internal component within the (macro) CNS: in any way, if the mutual manipulability criterion applies, because there is reciprocal causality between the parts.

## Footnotes

1 Since individual recognition is not necessary for social life, this line of reasoning does not apply to all social species. Invertebrates, for example, have not been fully investigated in this respect, presenting mixed evidence for either class recognition, kin recognition or familiar recognition, but never for the full set of properties defining true individual recognition (Gherardi et al. 2012).

2 “Information” is a difficult concept with multiple meanings. At least three broad conceptions of information can be distinguished: the statistical, the semantic/conventional, and the physical (Harms 2006). Throughout this paper we use information in the semantic/conventional sense, to characterise “knowledge of” or “meaning” as, for instance, witnessed in both the referential properties of symbols, and instructional aspects of knowledge in natural biological systems.

3 For example, well known generalists like the kea parrot *Nestor notabilis* (Young et al. 2012) are remarkable for presenting particularly high levels of technical intelligence (Huber and Gajdon 2006).

4 A proprietary demarcation criterion specifies the condition to be met for a process to count as cognitive. There are some candidate criteria to mark a process as cognitive, but the most widely endorsed is the “non-derived content”. For example, Adams and Aizawa (2001) propose that cognitive processes must involve the transformation and manipulation of representational states bearing non-derived contents, i.e., bearing contents that do not derive from the external world, that are thus intrinsic to the cognitive system. The problem here is that derived content is an ill defined concept, sometimes connected to intuitions about intentionality, without any consensus as how to differentiate derived from non-derived content. Also, a yet unavailable, consensual and complete theory of content would be necessary to account for the origin of these intrinsic, non-derived contents, within a naturalistic perspective (Shapiro 2009; Kaplan 2012; Gallagher 2013).

5 The bandwidth criterion relies on the pattern of causal interactions among the parts of a system. It states that causal connectivity should be stronger and more complex within the system, and weaker and simpler at the interfaces between distinct systems. Thus, the amount of connectivity should be used to decide the boundaries of the system.

6 It is important to note that these criticisms apply specifically to the approach put forward by Clark and Chalmers (1998) (see Clark 2006). Here, we are using the term ‘extended cognition’ in a broad sense to refer to any approach that proposes the extension of cognition to areas outside the brain, which include other approaches, such as the embodied and the enactivist ones.

7 This is not to say that web threads alone could in any way decide about the meaning of a vibration, as if the web was an extended phenotype that gained autonomy from the spider. As we emphasised above (note 1), the meaning of a piece of information is a systemic property, and we are thus stating that the web is part of the system that conveys meaning to the vibratory information. More than that, we are stating that the web processes information in adaptive ways, so as to impart meaning to it within the encompassing system. To make clearer this point, even the information that is passing in our optic lobes at any moment has no meaning in itself, without a connotative operation (sensu Harms 2004, 2006), an interpretation that occurs within the larger encompassing system, that relies on memory retrieval, on operations over these memories, and on emotional or activational states.

8 Nevertheless, there is experimental evidence for the centralised modulation of the most peripheral sensory neurons. Centrally released octopamine (Seyfarth et al. 1993) causes a persistent increase in excitability in spider mechanosensory neurons (Widmer et al. 2005), promoting a rise in sensitivity to higher frequencies (Torkkeli et al. 2011), and thus helping the spider to filter out irrelevant, low-frequency background noise. In this way, although the evidence available implies that the cognitive system does not extend to the matched filters themselves, it also implies that it does extend from the CNS to the periphery of the nervous system.
